# Trends in Comorbid Conditions Among Medicaid Enrollees With HIV

**DOI:** 10.1093/ofid/ofz124

**Published:** 2019-03-10

**Authors:** Megan B Cole, Omar Galárraga, Momotazur Rahman, Ira B Wilson

**Affiliations:** 1 Department of Health Law, Policy, and Management, Boston University School of Public Health, Boston, Massachusetts; 2 Department of Health Services, Policy, and Practice, Brown University School of Public Health, Providence, Rhode Island

**Keywords:** aging, comorbid conditions, HIV, Medicaid

## Abstract

**Background:**

As antiretroviral therapy has become more effective, persons with HIV live longer and develop conditions that are characteristic of older populations. Understanding changes in comorbid conditions has important implications for the complexity and cost of care, particularly for Medicaid programs and their enrollees, which comprise about 40% of all persons with HIV. Thus, our objective was to examine trends in comorbid conditions for Medicaid enrollees with HIV.

**Methods:**

Using 2001–2012 administrative claims data from the 14 states (NY, CA, FL, TX, MD, NJ, PA, IL, GA, NC, VA, LA, OH, MA) with the highest HIV prevalence, we identified 494 322 unique Medicaid enrollees with HIV, representing 5.8 million person-quarters after exclusions. We estimated changes over time in enrollee characteristics, proportions of enrollees with the 10 most common comorbid conditions, and number of comorbid conditions per enrollee.

**Results:**

Over time, the average age for HIV Medicaid enrollees increased, and the proportion enrolled in a managed care plan also increased. In 2012, the highest proportion of enrollees exhibited evidence of hypertension (31%), psychiatric disease (26%), any liver disease (25%), and pulmonary disorder (23%). Nine of the 10 comorbid conditions increased over time, whereas HIV-related conditions declined. The largest adjusted relative increases in 2012 vs 2003 were observed for renal insufficiency (adjusted odds ratio [aOR], 2.20; *P* < .001), hyperlipidemia (aOR, 1.80; *P* < .001), and psychiatric disease (aOR, 1.45; *P* < .001).

**Conclusions:**

Despite improvements in antiretroviral therapy and better control of patients’ HIV, we found substantial increases in rates of comorbid conditions over time. These findings have important implications for the complexity and costs of clinical care and for state Medicaid programs.

More than 40 000 new HIV infections occur every year in the United States [1, 2] and the number of prevalent cases of HIV was >1.1 million in 2013 [3]. Meanwhile, the advent of effective combination antiretroviral therapy (ART) in 1996 extended life expectancy for a 20-year-old person with HIV from 39 years in 1996 to 73 years in 2011 [4, 5]. As people with HIV live longer, they appear to develop diseases and conditions that are characteristic of middle-aged and older populations, including rising obesity and weight gain [6], diabetes, cardiovascular disease, and other chronic conditions [7]. Additionally, HIV and its treatment may themselves increase the prevalence of some conditions. Collectively, these noninfectious complications are referred to as HIV-associated non-AIDS (HANA) conditions [8–10]. HANA conditions have important implications for the complexity of providing quality clinical care given that such care often involves polypharmacy and multiple specialty providers. HANA conditions also have an economic impact, with the majority of the costs covered by Medicaid and Medicare [11].

Understanding disease burden in HIV-infected persons in the United States, and how this burden is changing over time, is challenging. Pioneering efforts have been made in the Veterans Aging Cohort Study (VACS) [12, 13], but there is little similar evidence from other nationally representative populations with large numbers of people living with HIV. Some efforts at the state level have helped our understanding of costs and care of individual conditions [14–16], but few studies have conducted multistate analyses [17–20]. One recent study examined trends in comorbidities for commercial, Medicaid, and Medicare enrollees with HIV, though the size and generalizability of the Medicaid population were limited [21]. No studies to our knowledge have been representative longitudinal studies, nor have any focused specifically on the Medicaid population.

Our objective was therefore to identify trends in HIV Medicaid enrollee characteristics and comorbidities over time. The HIV Medicaid population is important because Medicaid is the largest source of insurance coverage among persons with HIV, covering >40% of all persons with HIV who receive regular care [11]. Medicaid also covers some of the most vulnerable patients in the United States, as Medicaid enrollees are all low-income and are disproportionately disabled [22] or from racial/ethnic minority groups [23]. This represents the first known large-scale effort to characterize Medicaid enrollees living with HIV and associated comorbidities.

## METHODS

We used a retrospective study design using administrative claims data from 2001–2012 Medicaid Analytic eXtract (MAX) data from the 14 states with the highest HIV prevalence: New York, California, Florida, Texas, Maryland, New Jersey, Pennsylvania, Illinois, Georgia, North Carolina, Virginia, Louisiana, Ohio, and Massachusetts. These states accounted for about 75% of HIV prevalence in the United States in 2012 [24]. We obtained a 100% sample for each state.

### Sample Selection

We first identified a sample of n = 85 389 808 Medicaid enrollees for the years 2001–2012 from the 14 states. Second, we identified an initial HIV sample of n = 615 281 individuals using ICD-9 codes (HIV disease code 042, asymptomatic HIV code V08) or an indicator of ever having received ART. Third, from the initial sample, we classified enrollees into “HIV likely” vs “HIV possible” (see the flowchart in the Supplementary Figure A1). Those in the “HIV likely” category (n = 494 322) constituted our *base cohort* and included (1) those with ≥2 diagnostic codes for HIV or prescriptions for ≥2 antiretroviral medications (n = 448 284); (2) those with only 1 HIV diagnostic code from an inpatient or long-term care stay (n = 18 246); (3) those with only 1 diagnostic code for HIV but an additional diagnostic code for HIV wasting or HIV dementia (n = 6705); or (4) only 1 HIV diagnostic code but codes for ≥2 CD4 count tests (n = 21 087).

The *base cohort* of n = 494 322 individuals translated into a final *analytic sample* of n = 5 848 394 person-quarters. This sample excluded those not eligible for Medicaid for the full quarter (n = 1 920 742), those in comprehensive managed care in Massachusetts or Pennsylvania (n = 369 814) due to incomplete encounter data [25], those age <18 years (n = 481 718) or >64 years (n = 562 305) in the quarter, those dually eligible for Medicaid and Medicare in the quarter (n = 492 124), and quarters where an individual had <12 months of Medicaid eligibility in the look-back period (n = 552 333). An additional n = 606 694 person-quarters from 2001–2002 were excluded from rate calculations, as these person-quarters lacked sufficient prior observation time, though these quarters were used in classifying enrollees as having comorbid conditions.

### Measures

Our primary independent variable was an indicator for year. Our dependent variables included indicators for 10 of the most prevalent comorbid conditions observed in claims: cardiovascular disease, any pulmonary disorder, hypertension, diabetes, any liver disease, psychiatric disease, hyperlipidemia, anemia, renal insufficiency, and an indicator for having any HIV-related condition (see Supplementary Table A2 for detailed measure definitions). We also examined total number of comorbid conditions per enrollee out of 27 total comorbid conditions. To identify these comorbidities, we used the classification system developed by the VACS [26]. For each comorbid condition, we required 2 outpatient claims or 1 inpatient claim with evidence of the condition within the look-back period. The full list of conditions is presented in the Supplementary Data.

### Analyses

The unit of observation was the person-quarter. In our primary analysis, to identify evidence of a comorbid condition, we used a rolling 2-year look-back period. For a patient eligible for the full quarter *q*, if the patient had evidence of a comorbid condition in quarter *q* or in the preceding 7 quarters, then the patient was classified as having that condition in quarter *q.*

We calculated descriptive statistics by year to assess the changing demographics of the HIV Medicaid population. To calculate trends in comorbidities over time, for the 10 most common comorbidities, we used logistic models to estimate unadjusted and adjusted changes in the odds of an enrollee having each condition. To calculate trends in the number of comorbid conditions over time, we used linear regression models. For all models, we treated year as an indicator variable. Our logistic models estimated the odds of an enrollee having a condition in each year from 2004 to 2012 as compared with 2003. Our linear models estimated the mean additional number of comorbid conditions per enrollee in each year from 2004 to 2012 as compared with 2003. The years 2001 and 2002 were excluded from outcome analyses to allow for a 2-year look-back period, though data from these years were used in determining whether an enrollee had a condition in 2003. Adjusted models included age, sex, race, disability status, managed care enrollment, and state. Both adjusted and unadjusted models accounted for months of eligibility in the look-back period. For all analyses, we used robust variance estimators with 99% confidence intervals. The significance level was set at *P* < .001.

### Sensitivity Analyses

We conducted sensitivity analyses to ensure that results were robust. First, we estimated each outcome using an all-quarter look-back, where a patient eligible for the full quarter *q* was classified as having that condition in quarter *q* if the patient had claims evidence of a comorbid condition in quarter *q* or in *any* of the preceding quarters. Arguably, this all-quarter look-back method would more closely capture the concept of condition prevalence. Second, we repeated all analyses using a 1-year rolling look-back period. Third, we re-ran all primary analyses clustering errors at the state level in place of robust variance estimators. Finally, we re-ran our analyses examining changes in number of comorbid conditions using Poisson regression.

## RESULTS

### Trends in Population Demographics and Service Utilization

The characteristics of the HIV Medicaid population did not meaningfully change from 2003 to 2012 (Table 1), with 2 exceptions. The population grew older, from an average age of 40.5 years in 2003 to 43.4 years in 2012. Additionally, there was a large increase in the proportion of enrollees with comprehensive managed care (22%–58%). The likelihood of any utilization experienced small changes from 2003 to 2012, including a slight decrease in the percentage of enrollees with 1 or more inpatient-days per year (29%–25%).

**Table 1.** HIV Medicaid Enrollee Characteristics, 2003–2012

	2003	2004	2005	2006	2007	2008	2009	2010	2011	2012	All Years
Age, mean, y	40.5	40.9	41.2	41.7	42.0	42.2	42.3	42.7	43.0	43.4	42.0
Female, %	54	54	54	53	53	54	54	53	53	53	54
Race, %											
White, non-Hispanic	18	18	18	18	18	18	18	18	18	18	18
Black, non-Hispanic	53	53	52	52	51	51	51	51	51	51	52
Hispanic	20	20	21	21	21	21	21	21	21	21	21
Other	9	9	9	9	10	10	10	10	10	10	10
Disabled, %	60	59	58	58	58	57	56	57	57	56	58
Comprehensive managed care, %	22	25	27	29	32	35	37	40	49	58	36
1 or more claim in year, %	97	97	97	97	97	97	97	97	98	98	97
1+ IP day in year, %	29	29	29	29	28	27	27	26	26	25	28
1+ LT day in year, %	4	5	5	5	5	5	4	4	4	3	4
1+ OT day in year, %	97	97	97	97	97	97	97	97	97	98	97

n = 5 848 394 person-quarters. IP is inpatient care, LT is long-term care, and OT is other therapy, which includes physician services, lab/x-ray, clinic services, home health, hospice, and outpatient hospital institutional claims. Disabled implies that disability was the basis of eligibility for Medicaid.

### Trends in Comorbidities

When aggregating data from 2003 to 2012, hypertension was the most common comorbid condition (26.6%). This was followed by psychiatric disease (23.7%), any liver disease (22.6%), and pulmonary disorder (21.9%). At least 1 in 8 enrollees showed evidence of any HIV-related condition, cardiovascular disease, hyperlipidemia, and/or diabetes. Enrollees had a mean of 3.0 chronic conditions, and approximately 75% had evidence of at least 1 chronic condition. The full results are presented in the Supplementary Data.

**Figure 1.**
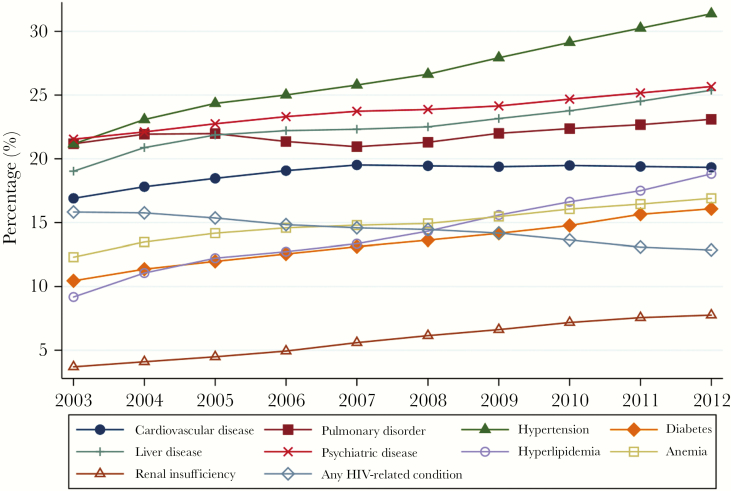
Rates of the top 10 most common comorbid conditions among HIV Medicaid enrollees, 2003–2012. n = 5 848 394 person-quarters. All yearly estimates have *P* values <.001. The denominator includes all eligible HIV Medicaid enrollees in the analytic cohort. The numerators for each condition include all patients who had evidence of the condition in the last 24 months, as documented in claims. Rates were adjusted for months of eligibility and not adjusted for patient characteristics.

**Figure 2.**
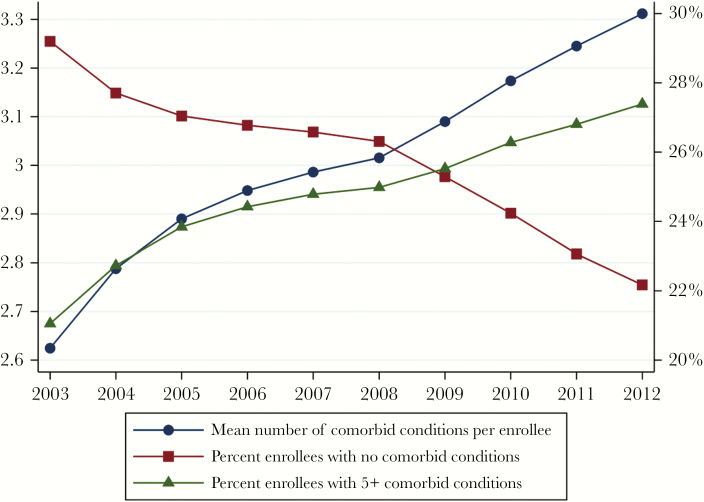
Number of comorbid conditions among HIV Medicaid enrollees, 2003–2012. n = 5 848 394 person-quarters. All yearly estimates have *P* values <.001. The denominator includes all eligible HIV Medicaid enrollees in the analytic cohort. The numerators include all patients who had evidence of the number of conditions in the last 24 months, as documented in claims. Rates were adjusted for months of eligibility and not adjusted for patient characteristics. The left y-axis represents the mean number of comorbid conditions/enrollee, and the right y-axis represents the percentage of enrollees with either 0 or 5+ comorbid conditions.

Unadjusted changes over time for the 10 most common conditions are shown in Figure 1. Nine of the 10 conditions increased over time, with only HIV-related conditions declining; though not among the top 10 conditions, rates of HIV-related cancer also declined, whereas rates of all other cancer increased (Supplementary Data). As shown in Figure 2, the percentage of enrollees exhibiting no evidence of any of the 27 measured comorbid conditions decreased from 29.2% in 2003 to 22.2% in 2012, whereas the percentage of enrollees exhibiting evidence of 5 or more conditions increased from 21.1% in 2003 to 27.4% in 2012—indicating that more than one-quarter of HIV Medicaid enrollees had 5 or more comorbid conditions by the end of the study period.

Table 2 shows the unadjusted and adjusted odds ratios (aORs) of having the condition in 2012 as compared with 2003. Conditions showing the greatest adjusted relative increases between 2003 and 2012 using the 2-year look-back (Table 2, column 1) were renal insufficiency (3.7% in 2003 to 7.8% in 2012; adjusted odds ratio [aOR], 2.20), hyperlipidemia (9.2%–18.8%; aOR, 1.80), psychiatric disease (21.5%–25.7%; aOR, 1.45), anemia (12.3%–16.9%; aOR, 1.38), hypertension (21.2%–31.3%; aOR, 1.31), and liver disease (19.0%–25.4%; aOR, 1.31). The largest unadjusted increases over time were observed for hyperlipidemia (odds ratio [OR], 2.30), renal insufficiency (OR, 2.20), hypertension (OR, 1.72), and diabetes (OR, 1.65). Having at least 1 HIV-related condition was the only outcome to decrease (15.8% in 2003 to 12.9% in 2012; aOR, 0.82). The number of comorbid conditions per patient increased from 2.62 to 3.31. Details are shown in the Supplementary Data.

**Table 2.** Changes in Comorbidities Among HIV Medicaid Enrollees, 2012 vs 2003

	Primary Analysis				Sensitivity Analyses							
	2-Year Look-Back				1-Year Look-Back				All-Year Look-Back						
	2003	2012	OR	aOR	2003	2012	OR	aOR	2003	2012	OR	aOR
Hypertension	21.2%	31.3%	1.72	1.31	17.5%	25.9%	1.64	1.40	22.7%	38.3%	2.16	1.71
Psychiatric disease	21.5%	25.7%	1.26	1.45	16.8%	19.5%	1.19	1.53	23.7%	41.2%	2.33	2.74
Liver disease	19.0%	25.4%	1.45	1.31	15.1%	20.5%	1.45	1.36	20.3%	35.2%	2.16	2.05
Pulmonary disorder	21.2%	23.1%	1.12	1.05	16.5%	17.4%	1.07	1.13	23.1%	33.3%	1.68	1.63
Cardiovascular disease	16.9%	19.3%	1.18	1.03	12.8%	14.0%	1.11	1.09	18.6%	30.4%	1.94	1.75
Hyperlipidemia	9.2%	18.8%	2.30	1.80	6.9%	14.1%	2.20	1.89	10.0%	26.7%	3.33	2.65
Any HIV-related condition	15.8%	12.9%	0.78	0.82	11.5%	8.6%	0.72	0.85	17.7%	23.2%	1.42	1.48
Anemia	12.3%	16.9%	1.45	1.38	9.1%	11.9%	1.35	1.42	13.6%	28.2%	2.51	2.36
Diabetes	10.5%	16.1%	1.65	1.26	9.0%	13.4%	1.56	1.30	11.0%	20.3%	2.07	1.62
Renal insufficiency	3.7%	7.8%	2.20	2.20	2.9%	5.9%	2.08	2.12	4.0%	11.7%	3.22	3.18
	2003	2012	Coeff.	Adj	2003	2012	Coeff.	Adj	2003	2012	Coeff.	Adj
				Coeff.				Coeff.				Coeff.
No. of comorbid conditions	2.62	3.31	0.69	0.58	2.05	2.47	0.42	0.22	2.83	5.25	2.43	2.24

n = 5 848 394 person-quarters. The denominator includes all eligible HIV Medicaid enrollees with at least 12 months of eligibility in the look-back period. The numerators for each condition include all patients who had evidence of the condition, as documented in claims. Rates from 2003 and 2012 are unadjusted for patient characteristics. OR is odds ratio and aOR is adjusted odds ratio, as derived from the logistic regression model, where an OR > 1 implies that a greater proportion of enrollees had evidence of that condition in 2012 as compared with 2003. Coeff is coefficient, as derived from the linear regression model, where a coeff > 0 implies that enrollees had more comorbid conditions in 2012 as compared with 2003. Adjusted estimates were adjusted for age, sex, race, disability status, managed care status, state, and months of eligibility in the look-back period. Unadjusted estimates were only adjusted for months of eligibility in the look-back period. All estimates have *P* values <.001. For confidence intervals, see the Supplementary Data.

### Sensitivity Analyses

Results from all sensitivity analyses were directionally similar to our primary analyses. As anticipated, more conservative assumptions yielded smaller effect sizes, and less conservative assumptions yielded larger effect sizes. As shown in Table 2 (third set of columns), when using an all-quarter look-back period, rates for all conditions were higher in 2012 as compared with 2012 using the 2-year look-back period. For instance, the all-quarter look-back shows that about 40% of enrollees had evidence of hypertension or psychiatric disease by 2012, and about one-third had pulmonary disorder, liver disease, and/or cardiovascular disease by 2012. Adjusted regression estimates suggest that for half of the conditions examined, the odds of an enrollee having evidence of that condition more than doubled between 2003 and 2012. Additional sensitivity analyses are shown in the Supplementary Data.

## DISCUSSION

From 2001 to 2012, we found that rates of comorbid conditions in Medicaid enrollees with HIV increased notably. The overall burden of comorbid disease was substantial; in 2012, the mean number of comorbid conditions, summing across the 27 conditions that we that we measured, was 3.3, with over one-quarter of the population showing evidence of 5 or more conditions. The most prevalent conditions in 2012 were hypertension, psychiatric disease, liver disease, and pulmonary disorder, all with rates >20%, and all-time look-back results suggesting rates exceeding 30% by 2012. Furthermore, demographic composition of Medicaid enrollees with HIV changed only slightly over time, with enrollees becoming older and more likely to have managed care. However, trends persisted after adjustment for these and other demographic factors.

Although rates of comorbid conditions increased over time, the percentage of enrollees having any HIV-related condition declined; this was also true of HIV-related cancers. This is likely due to dramatic improvements in ART. The most consequential improvement was widespread implementation of 1-pill-once-a-day therapy, but there is also evidence that ART adherence improved over this time period [27]. As would be expected if virologic control improved, our data show that rates of HIV-related conditions fell, though rates of all HANA conditions increased.

The conditions with the largest unadjusted increases over time included hyperlipidemia, hypertension, and diabetes—chronic conditions associated with older age but not specifically associated with HIV [7, 28, 29]. Although these data do not imply that increased rates are caused by HIV or its treatments, they do suggest that these age-associated conditions may be increasing at faster rates in persons with HIV, when compared with the literature on trends in the general US population [30–32].

Although anemia has historically been associated with HIV clinical disease progression, it is difficult to speculate on why rates of anemia are increasing given that these claims data do not include laboratory diagnoses. The etiology of anemia in HIV is clearly multifactorial [33]. One of the most intriguing hypotheses is that HIV, even when treated, is a proinflammatory state that looks a great deal like the anemia of inflammation often seen in aging populations that may be driven by factors such as abnormal cytokine production and disordered iron metabolism [34].

This is the first large, multistate longitudinal study of comorbid conditions in Medicaid enrollees with HIV that has documented changes in rates of comorbid conditions over a 10-year period, covering approximately 75% of HIV Medicaid patients in the United States. Only a few prior studies have examined changes over time in rates of comorbid conditions in persons with HIV, none of which have focused explicitly on the Medicaid population. Gallant et al. examined trends in comorbidities for commercial, Medicaid, and Medicare enrollees with HIV and found increases in comorbid conditions [21]. However, the Medicaid population examined was limited to 26 000 HIV-infected enrollees, and analyses did not account for critical Medicaid-specific factors, such as dual eligibility, managed care enrollment, or basis of eligibility. Erdem et al. examined Medicare Part A beneficiaries with HIV and found that chronic condition rates increased between 2008 and 2010 [35]. In an Italian HIV cohort that directly examined patients, rates of hypertension and diabetes increased between 2010 and 2014 [36]. A Swiss HIV cohort study analyzed rates of comorbid conditions in 2008 by direct examination, as well as rates of clinical events in the subsequent 3 years, but did not examine longitudinal changes [37]. A 2007 VACS study examined comorbidities in veterans with HIV [26]. However, differing demographics of veteran and Medicaid populations limit the value of direct comparisons, and we are not aware of data from the VACS cohort that describe changes in prevalence of comorbid conditions over time.

It is important to note that this is not a classic cohort study or a balanced panel because people entered and left the study sample every month as they gained and lost Medicaid coverage. However, all Medicaid enrollees that we studied were Medicaid eligible for at least 1 continuous year, and the average enrollee was eligible for nearly 75% of all months in the study period. Thus, our analysis has features of both a cohort study and a serial, cross-sectional analysis. The population examined is the complete 100% universe of Medicaid patients in 14 states over the 12-year period. Thus, changes in rates of comorbid conditions can be interpreted as a measure of the increased burden of illness that Medicaid providers are facing over that time, and not as a pure measure of epidemiologic trend or sample anomaly.

Although we use a rolling 2-year look-back period to control for observation time bias when estimating time trends, our sensitivity analyses using an all-quarter look-back period may more accurately capture the concept of point-in-time disease prevalence, particularly for later years of the study period. These estimates suggest an even greater burden of disease in the HIV Medicaid population than observed in our primary analyses.

Our findings have 2 main implications for clinicians and policy-makers. First, because Medicaid patients with HIV are aging and accumulating comorbid conditions, their care is increasingly complex. Persons with HIV will gradually need primary care, HIV care, *and* other specialty care, which may involve coordination among multiple providers. Polypharmacy and its attendant challenges will also increase as enrollees take more medications for multiple conditions [38]. Thus, it is important for Medicaid providers and health plans to ensure access to high-quality coordinated care for this population while accounting for increasingly complex patient needs through appropriate disease management.

Second, notwithstanding ways in which effective ART, if used correctly, can prevent progression of HIV, the care of persons with HIV is likely to become costlier. These costs are disproportionately borne by the public sector. About 25% of persons with HIV have Medicare coverage, most of whom are dually eligible for Medicaid [39], and about 40% have Medicaid coverage [11]. In 2011, per capita spending on HIV Medicaid enrollees was $26 807—approximately 5 times that of the average Medicaid enrollee [11]. Meanwhile, in recent years, Congress has been considering changes to the Affordable Care Act, including to the Medicaid program. As our findings show, persons with HIV are increasingly complex, and thus expensive, to care for. Legislation that reduces access to care for persons with HIV would have severely negative effects on HIV patients [40]. Our findings underline the importance of access to and affordability of care for persons with HIV, particularly given that not only HIV infection but also most of the comorbid conditions examined are highly treatable.

### Limitations

This analysis has limitations. First, we were unable to estimate true disease prevalence given that we only observed what was present in claims; thus, we instead captured evidence of service utilization for comorbid conditions. We used rolling quarters to capture this service utilization, to minimize observation time bias, but this likely resulted in conservative estimates of true underlying prevalence. Second, claims data were limited in that they were used for billing purposes and lacked clinical indicators. This may have resulted in misclassification, which would have underestimated rates of comorbid conditions. This also means that we were unable to completely capture the etiology of disease and that some important conditions, such as nondementing cognitive decline, were not well captured in the data. Third, the results rely on reports from individual state Medicaid programs, which do not have consistent reporting and coding procedures. Although a claims aggregator was used to standardize data, there may have been state anomalies that affected aggregate rates even after adjusting for state. Fourth, we did not exclude patients in managed care plans because MAX files included reliable encounter data from health plans in most states [41], except for Massachusetts and Pennsylvania, which were excluded. To account for reporting and population differences, we controlled for managed care in our models, though this may still have underestimated disease prevalence, particularly in later years when managed care participation is greater. Fifth, because we only observed patients enrolled in Medicaid, we did not have data on the date of first HIV diagnosis, which may predate enrollment in Medicaid; thus we could not account for duration of diagnosis. Finally, although the 14 states examined capture ~75% of HIV prevalence, the results may not be generalizable to all 50 states.

### Conclusions

In conclusion, from 2001 to 2012, when there were dramatic improvements in ART and ART adherence, our data showed corresponding declines in rates of HIV-related conditions over this time period. However, notwithstanding these improvements in HIV patients’ primary disease, there were substantial increases in rates of comorbid conditions. By 2012, rates of hypertension, psychiatric disease, liver disease, and pulmonary disorder were all >20%, and enrollees had about 3.1 comorbid conditions on average, with over a quarter of enrollees having 5 or more conditions. These findings have important implications for the complexity and costs of clinical care and for the state Medicaid programs serving these patients.

## Supplementary Data

Supplementary materials are available at *Open Forum Infectious Diseases* online. Consisting of data provided by the authors to benefit the reader, the posted materials are not copyedited and are the sole responsibility of the authors, so questions or comments should be addressed to the corresponding author.

Supplementary Material

## References

[1] Hall HI, Song R, Rhodes P, et al; HIV Incidence Surveillance Group Estimation of HIV incidence in the United States. JAMA 2008; 300:520–9. 18677024 10.1001/jama.300.5.520 PMC2919237

[2] Prejean J, Song R, Hernandez A, et al; HIV Incidence Surveillance Group Estimated HIV incidence in the United States, 2006-2009. PLoS One 2011; 6:e17502. 21826193 10.1371/journal.pone.0017502 PMC3149556

[3] Song R, Hall HI, Green TA, et al. Using CD4 data to estimate HIV incidence, prevalence, and percent of undiagnosed infections in the United States. J Acquir Immune Defic Syndr 2017; 74:3–9. 27509244 10.1097/QAI.0000000000001151

[4] Marcus JL, Chao CR, Leyden WA, et al. Narrowing the gap in life expectancy between HIV-infected and HIV-uninfected individuals with access to care. J Acquir Immune Defic Syndr 2016; 73:39–46. 27028501 10.1097/QAI.0000000000001014 PMC5427712

[5] Samji H, Cescon A, Hogg RS, et al; North American AIDS Cohort Collaboration on Research and Design (NA-ACCORD) of IeDEA Closing the gap: increases in life expectancy among treated HIV-positive individuals in the United States and Canada. PLoS One 2013; 8:e81355. 24367482 10.1371/journal.pone.0081355 PMC3867319

[6] Koethe JR, Jenkins CA, Lau B, et al; North American AIDS Cohort Collaboration on Research and Design (NA-ACCORD) Rising obesity prevalence and weight gain among adults starting antiretroviral therapy in the United States and Canada. AIDS Res Hum Retroviruses 2016; 32:50–8. 26352511 10.1089/aid.2015.0147 PMC4692122

[7] Scherer PE, Hill JA Obesity, diabetes, and cardiovascular diseases: a compendium. Circ Res 2016; 118:1703–5. 27230636 10.1161/CIRCRESAHA.116.308999 PMC4888905

[8] Torres RA, Lewis W Aging and HIV/AIDS: pathogenetic role of therapeutic side effects. Lab Invest 2014; 94:120–8. 24336070 10.1038/labinvest.2013.142 PMC4144856

[9] Deeks SG, Phillips AN HIV infection, antiretroviral treatment, ageing, and non-AIDS related morbidity. BMJ 2009; 338:a3172. 19171560 10.1136/bmj.a3172

[10] Guaraldi G, Orlando G, Zona S, et al. Premature age-related comorbidities among HIV-infected persons compared with the general population. Clin Infect Dis 2011; 53:1120–6. 21998278 10.1093/cid/cir627

[11] Kaiser Family Foundation. Medicaid and HIV 2016 http://kff.org/hivaids/fact-sheet/medicaid-and-hiv/. Accessed 15 June 2018.

[12] Gandhi NR, Tate JP, Rodriguez-Barradas MC, et al. Validation of an algorithm to identify antiretroviral-naïve status at time of entry into a large, observational cohort of HIV-infected patients. Pharmacoepidemiol Drug Saf 2013; 22:1019–25. 23836591 10.1002/pds.3476 PMC3831617

[13] Justice AC, Dombrowski E, Conigliaro J, et al. Veterans Aging Cohort Study (VACS): overview and description. Med Care 2006; 44(8 Suppl 2):S13–24. 16849964 10.1097/01.mlr.0000223741.02074.66 PMC3049942

[14] Leibowitz AA, Desmond K Identifying a sample of HIV-positive beneficiaries from Medicaid claims data and estimating their treatment costs. Am J Public Health 2015; 105:567–74. 25602870 10.2105/AJPH.2014.302263 PMC4324128

[15] Walkup J, Crystal S, Sambamoorthi U Schizophrenia and major affective disorder among Medicaid recipients with HIV/AIDS in New Jersey. Am J Public Health 1999; 89:1101–3. 10394325 10.2105/ajph.89.7.1101 PMC1508849

[16] Chesnut TJ, Laufer FN, Carrascal AF, Feldman IS An expenditure analysis of high-cost Medicaid recipients with HIV disease in New York State. J Health Care Poor Underserved 2011; 22:330–45. 21317526 10.1353/hpu.2011.0017

[17] Morin SF, Sengupta S, Cozen M, et al. Responding to racial and ethnic disparities in use of HIV drugs: analysis of state policies. Public Health Rep 2002; 117:263–72; discussion 231–2. 12432137 10.1016/S0033-3549(04)50160-7 PMC1497438

[18] Kahn JG, Zhang X, Cross LT, et al. Access to and use of HIV antiretroviral therapy: variation by race/ethnicity in two public insurance programs in the U.S. Public Health Rep 2002; 117:252–62; discussion 231–2. 12432136 10.1016/S0033-3549(04)50159-0 PMC1497435

[19] Johnston SS, Juday T, Seekins D, et al. Patterns and correlates of linkage to appropriate HIV care after HIV diagnosis in the US Medicaid population. Sex Transm Dis 2013; 40:18–25. 23250298 10.1097/OLQ.0b013e3182782014

[20] Johnston SS, Juday T, Farr AM, et al. Comparison between guideline-preferred and nonpreferred first-line HIV antiretroviral therapy. Am J Manag Care 2014; 20:448–55. 25180433

[21] Gallant J, Hsue PY, Shreay S, Meyer N Comorbidities among US patients with prevalent HIV infection-a trend analysis. J Infect Dis 2017; 216:1525–33. 29253205 10.1093/infdis/jix518

[22] Kaiser Family Foundation. Medicaid Pocket Primer – Fact Sheet Menlo Park, CA: Kaiser Family Foundation; 2017 http://files.kff.org/attachment/Fact-Sheet-Medicaid-Pocket-Primer. Accessed 15 June 2018.

[23] US Department of Commerce, Economics and Statistics Administration, United State Census Bureau. Health Insurance Coverage in the United States. Current Population Reports. Washington, DC: US Government Printing Office; 2017.

[24] Centers for Disease Control and Prevention. *HIV/AIDS Surveillance Report 2012.* Vol. 24. Atlanta, GA: Centers for Disease Control and Prevention; 2013.

[25] Centers for Medicaid and Medicare Services. *Managed Care Crosswalk - MAX 2009*. Baltimore, MD: Centers for Medicare and Medicaid Services; 2010.

[26] Goulet JL, Fultz SL, Rimland D, et al. Aging and infectious diseases: do patterns of comorbidity vary by HIV status, age, and HIV severity? Clin Infect Dis 2007; 45:1593–601. 18190322 10.1086/523577 PMC3687553

[27] Youn B, Shireman TI, Lee Y, et al. Ten-year trends in antiretroviral therapy persistence among US Medicaid beneficiaries. AIDS 2017; 31:1697–707. 28700393 10.1097/QAD.0000000000001541 PMC5625296

[28] Félix-Redondo FJ, Grau M, Fernández-Bergés D Cholesterol and cardiovascular disease in the elderly. Facts and gaps. Aging Dis 2013; 4:154–69. 23730531 PMC3660125

[29] Mozaffarian D, Benjamin EJ, Go AS, et al; American Heart Association Statistics Committee and Stroke Statistics Subcommittee Heart disease and stroke statistics–2015 update: a report from the American Heart Association. Circulation 2015; 131:e29–322. 25520374 10.1161/CIR.0000000000000152

[30] Centers for Disease Control, Division of Diabetes Translation. Long-term Trends in Diabetes. Atlanta, GA: Centers for Disease Control; 2017.

[31] Rosinger A, Carroll MD, Lacher D, Ogden C Trends in total cholesterol, triglycerides, and low-density lipoprotein in US adults, 1999–2014. JAMA Cardiol 2017; 2:339–41. 27902824 10.1001/jamacardio.2016.4396 PMC7388068

[32] Zhang Y, Moran AE Trends in the prevalence, awareness, treatment, and control of hypertension among young adults in the United States, 1999 to 2014. Hypertension 2017; 70:736–742. 28847890 10.1161/HYPERTENSIONAHA.117.09801 PMC5657525

[33] Redig AJ, Berliner N Pathogenesis and clinical implications of HIV-related anemia in 2013. Hematology Am Soc Hematol Educ Program 2013; 2013:377–81. 24319207 10.1182/asheducation-2013.1.377

[34] Vanasse GJ, Berliner N Anemia in elderly patients: an emerging problem for the 21^st^ century. Hematology Am Soc Hematol Educ Program 2010; 2010:271–5. 21239805 10.1182/asheducation-2010.1.271

[35] Erdem E Prevalence of chronic conditions among Medicare Part A beneficiaries in 2008 and 2010: are Medicare beneficiaries getting sicker? Prev Chronic Disease 2014; 11: E10. 10.5888/pcd11.130118 PMC3899849 24433626

[36] De Socio GV, Ricci E, Maggi P, et al; CISAI Study Group Time trend in hypertension prevalence, awareness, treatment, and control in a contemporary cohort of HIV-infected patients: the HIV and Hypertension Study. J Hypertens 2017; 35:409–16. 28005710 10.1097/HJH.0000000000001150

[37] Hasse B, Ledergerber B, Furrer H, et al; Swiss HIV Cohort Study Morbidity and aging in HIV-infected persons: the Swiss HIV cohort study. Clin Infect Dis 2011; 53:1130–9. 21998280 10.1093/cid/cir626

[38] Edelman EJ, Gordon KS, Glover J, et al. The next therapeutic challenge in HIV: polypharmacy. Drugs Aging 2013; 30:613–28. 23740523 10.1007/s40266-013-0093-9 PMC3715685

[39] Kaiser Family Foundation. Medicare and HIV 2016 http://kff.org/hivaids/fact-sheet/medicare-and-hiv/. Accessed 15 June 2018.

[40] Dawson L, Kates J; Kaiser Family Foundation What is at stake in ACA repeal and replace for people with HIV? http://kff.org/hivaids/issue-brief/what-is-at-stake-in-aca-repeal-and-replace-for-people-with-hiv/. Accessed 15 June 2018.

[41] Byrd VLH, Dodd AH, Malsberger R, Zlatinov A; Mathematica Policy Research. Assessing the usability of MAX 2008 encounter data for enrollees in comprehensive managed care. Brief 7. 2012 https://www.cms.gov/Research-Statistics-Data-and-Systems/Computer-Data-and-Systems/MedicaidDataSourcesGenInfo/Downloads/MAX_IB7_EncounterData_071312.pdf. Accessed 15 June 2018.

