# Supplementary material for: Trends in Comorbid Conditions Among Medicaid Enrollees With HIV
Source: Open Forum Infect Dis. 2019 Mar 10;6(4):ofz124. doi: 10.1093/ofid/ofz124 (PMC6453520; doi:10.1093/ofid/ofz124)
Supplement: Supplementary Material [file ofz124_suppl_supplementary_material.docx]

**SUPPLEMENTAL CONTENT**

Table of Contents

[SUPPLEMENTAL CONTENT A: SAMPLE SELECTION 2](#_Toc527987297)

[SUPPLEMENTAL CONTENT B: MEASURE DEFINITIONS 3](#_Toc527987298)

[SUPPLEMENTAL CONTENT C: PRIMARY ANALYSIS, FULL RESULTS 7](#_Toc527987299)

[SUPPLEMENTAL CONTENT D: ADDITIONAL SENSITIVITY ANALYSES 13](#_Toc527987300)

# SUPPLEMENTAL CONTENT A: SAMPLE SELECTION

**Figure A1.** Base sample of Medicaid enrollees likely living with HIV in 14 states, 2001-2012

Enrollees with Medicaid claims (n=85,389,808)

At least 1 ART Rx or 1 HIV Dx (n=615,281)

Only one HIV Dx (n=166,520)

Single HIV Dx from OT (n=148,274)

No HIV Wasting or Dementia Dx (n=120,482)

**HIV likely:**

at least

2 CD4 count

tests

(n=21,087)

**HIV likely:**

Have

at least

one HIV

wasting/

dementia Dx

(n=6,705)

**HIV possible:** Have no HIV related Dx or <2 CD4 count test (n=120,482)

**HIV likely:** Single HIV dx from LT or IP (n=18,246)

**HIV likely:** At least 2+HIV Dx or 2+ ART ingredients (n=448,284)

**HIV possible:** only one ART ingredient Rx (n=53) or only one ART ingredient Rx and one HIV OT Dx (n=424)

Total n for HIV likely

n=494,322

Total n for HIV possible

(excluded)

n=120,959

**Notes**: n refers to individual Medicaid enrollees; ART is antiretroviral therapy; Dx is diagnosis; Rx is prescription filled; IP is inpatient; LT is long-term care; OT is other care (including outpatient and professional services).

# SUPPLEMENTAL CONTENT B: MEASURE DEFINITIONS

**Table A1.** Percent of HIV Medicaid Enrollees Exhibiting Evidence of Comorbid Conditions – Full List

| **Condition** | **Mean, 2003-2012** |
| --- | --- |
| Any drug abuse+ | 28.4% |
| Hypertension | 26.6% |
| Psychiatric disease (any) | 23.7% |
| *Major depression* | 15.3% |
| *Other depression* | 15.3% |
| *Bipolar disorder* | 10.8% |
| *Schizophrenia* | 6.2% |
| *Schizoaffective disorder* | 6.4% |
| *Post-traumatic stress disorder* | 3.0% |
| *Other psychosis* | 8.5% |
| Liver disease (any) | 22.6% |
| *Any hepatitis C* | 18.7% |
| *Any hepatitis B* | 12.2% |
| *Decompensated liver disease* | 2.3% |
| *End stage liver disease* | 19.8% |
| Pulmonary disorder (any)^ | 21.9% |
| Cardiovascular disease (any) | 18.9% |
| *Myocardial infarction/Coronary artery disease* | 6.3% |
| *Congestive heart failure* | 12.4% |
| *Peripheral vascular disease* | 12.5% |
| Most anemias | 15.0% |
| HIV-related condition (any) | 14.4% |
| Hyperlipidemia | 14.2% |
| Diabetes mellitus (all types) | 13.4% |
| Any alcohol abuse | 10.1% |
| Renal insufficiency (any) | 5.8% |
| Pancreatitis | 4.1% |
| Cancer |  |
| *HIV-related** | 2.7% |
| *All other cancer* | 3.8% |
| Stroke (any) | 3.6% |
| Bone disorder** | 3.4% |
| Urine/kidney stone(s) | 2.8% |

**Notes:** Percentages represent aggregate 2003-2012 unadjusted means using a two-year look-back period to classify an enrollee as having a condition; n= 5,848,394 person-quarters

+ Although “any drug abuse” is among the top most prevalent conditions, we did not include this condition in our top 10 list.

^ Pulmonary disorder includes chronic obstructive lung disease, pulmonary hypertension, asthma, bronchiectasis, alveolitis, pneumoconiosis, and interstitial and/or fibrotic lung diseases.

*HIV-related cancers include Kaposi sarcoma, aggressive B-cell non-Hodgkin lymphoma, and cervical cancer.

**Bone disorder is inclusive of osteoporosis, osteopenia, and other bone disorders (ICD-9 733.*).

**Table A2.** Conditions included within “Any HIV-related Condition” Measure

| **Condition** |
| --- |
| 1. Kaposi’s sarcomas |
| 1. Pneumocystis pneumonia |
| 1. Tuberculosis |
| 1. Mycobacterium |
| 1. Cytomegalovirus |
| 1. Wasting |
| 1. Dementia* |
| 1. Candidiasis |
| 1. Cryptococciosis |
| 1. Histoplasmosis |
| 1. Isosporiasis |
| 1. Herpes zoster |
| 1. Visceral herpes simplex |
| 1. Bacterial pneumonia |

*For disaggregated trends of HIV-related dementia, see Table A9.

**Table A3.** 27 Conditions included within “Number of Comorbid Conditions” Measure

| **Condition** |
| --- |
| 1. Congestive heart failure |
| 1. Pulmonary disorder (any) |
| 1. Peripheral vascular disease |
| 1. Hypertension |
| 1. Diabetes mellitus (all types) |
| 1. Myocardial infarction/Coronary artery disease |
| 1. Pancreatitis |
| 1. Schizophrenia |
| 1. Schizoaffective disorder |
| 1. Other psychosis |
| 1. Bipolar disorder |
| 1. Major depression |
| 1. Other depression |
| 1. Post-traumatic stress disorder |
| 1. Stroke (any) |
| 1. Renal insufficiency (any) |
| 1. Any hepatitis B |
| 1. Any hepatitis C |
| 1. Most anemias |
| 1. Hyperlipidemia |
| 1. Urine/kidney stone(s) |
| 1. Cirrhosis of the liver |
| 1. Decompensated liver disease |
| 1. End-stage liver disease |
| 1. Any drug abuse |
| 1. Any alcohol abuse |
| 1. Any cancer |

# SUPPLEMENTAL CONTENT C: PRIMARY ANALYSIS, FULL RESULTS

**Table A4.** Changes in Comorbidities Amongst HIV Medicaid Enrollees Complete with Standard Errors, 2012 vs 2003

|  | **Unadjusted** | | | | | | **Adjusted^a^** | | | | | |
| --- | --- | --- | --- | --- | --- | --- | --- | --- | --- | --- | --- | --- |
|  | **OR** | **Robust SE** | **z** | **p>\|z\|** | **[99% Conf.** | **Interval]** | **OR** | **Robust SE** | **z** | **p>\|z\|** | **[99% Conf.** | **Interval]** |
| **Cardiovascular disease** | 1.18 | 0.01 | 33.96 | 0.000 | 1.17 | 1.20 | 1.03 | 0.01 | 5.19 | 0.000 | 1.01 | 1.04 |
| **Pulmonary disorder (any)** | 1.12 | 0.01 | 24.95 | 0.000 | 1.11 | 1.13 | 1.05 | 0.01 | 11.23 | 0.000 | 1.04 | 1.07 |
| **Hypertension** | 1.72 | 0.01 | 124.50 | 0.000 | 1.70 | 1.74 | 1.31 | 0.01 | 55.70 | 0.000 | 1.29 | 1.32 |
| **Diabetes** | 1.65 | 0.01 | 88.35 | 0.000 | 1.62 | 1.67 | 1.26 | 0.01 | 39.06 | 0.000 | 1.25 | 1.28 |
| **Liver disease (any)** | 1.45 | 0.01 | 81.74 | 0.000 | 1.43 | 1.47 | 1.31 | 0.01 | 55.54 | 0.000 | 1.29 | 1.33 |
| *Hepatitis C* | 1.16 | 0.01 | 29.97 | 0.000 | 1.14 | 1.17 | 1.10 | 0.01 | 18.16 | 0.000 | 1.08 | 1.11 |
| *Hepatitis B* | 1.34 | 0.01 | 49.22 | 0.000 | 1.32 | 1.36 | 1.17 | 0.01 | 24.93 | 0.000 | 1.15 | 1.19 |
| *Decompensated liver disease* | 1.21 | 0.02 | 14.62 | 0.000 | 1.17 | 1.25 | 1.10 | 0.01 | 6.98 | 0.000 | 1.06 | 1.14 |
| *End stage liver disease* | 1.01 | 0.00 | 2.18 | 0.030 | 1.00 | 1.02 | 1.09 | 0.01 | 17.87 | 0.000 | 1.08 | 1.11 |
| **Psychiatric disease (any)** | 1.26 | 0.01 | 52.44 | 0.000 | 1.25 | 1.28 | 1.45 | 0.01 | 79.17 | 0.000 | 1.43 | 1.47 |
| *Major depression* | 1.17 | 0.01 | 30.07 | 0.000 | 1.15 | 1.19 | 1.24 | 0.01 | 38.74 | 0.000 | 1.22 | 1.25 |
| *Other depression* | 1.04 | 0.01 | 7.36 | 0.000 | 1.02 | 1.05 | 1.14 | 0.01 | 23.69 | 0.000 | 1.12 | 1.15 |
| *Bipolar disorder* | 1.87 | 0.01 | 99.63 | 0.000 | 1.84 | 1.90 | 2.20 | 0.01 | 121.36 | 0.000 | 2.17 | 2.24 |
| *Schizophrenia* | 1.06 | 0.01 | 7.01 | 0.000 | 1.04 | 1.08 | 1.12 | 0.01 | 13.47 | 0.000 | 1.09 | 1.14 |
| *Schizoaffective disorder* | 1.17 | 0.01 | 20.36 | 0.000 | 1.15 | 1.19 | 1.33 | 0.01 | 35.13 | 0.000 | 1.30 | 1.36 |
| *PTSD* | 2.13 | 0.02 | 66.45 | 0.000 | 2.07 | 2.19 | 2.28 | 0.03 | 70.61 | 0.000 | 2.22 | 2.35 |
| *Other psychosis* | 1.46 | 0.01 | 55.25 | 0.000 | 1.44 | 1.49 | 1.79 | 0.01 | 81.55 | 0.000 | 1.76 | 1.83 |
| **Hyperlipidemia** | 2.30 | 0.01 | 145.52 | 0.000 | 2.27 | 2.34 | 1.80 | 0.01 | 97.59 | 0.000 | 1.77 | 1.83 |
| **Anemia (most)** | 1.45 | 0.01 | 69.57 | 0.000 | 1.43 | 1.47 | 1.38 | 0.01 | 57.45 | 0.000 | 1.36 | 1.40 |
| **Renal insufficiency (any)** | 2.20 | 0.02 | 91.25 | 0.000 | 2.15 | 2.25 | 2.20 | 0.02 | 88.02 | 0.000 | 2.15 | 2.25 |
| **Any HIV-related condition** | 0.78 | 0.00 | -45.73 | 0.000 | 0.77 | 0.79 | 0.82 | 0.00 | -35.62 | 0.000 | 0.81 | 0.83 |
|  | **Coef.** | **Robust SE** | **z** | **p>\|z\|** | **[99% Conf.** | **Interval]** | **Coef.** | **Robust SE** | **z** | **p>\|z\|** | **[99% Conf.** | **Interval]** |
| **No. of comorbid conditions** | 0.69 | 0.01 | 114.56 | 0.000 | 0.67 | 0.70 | 0.58 | 0.01 | 102.80 | 0.000 | 0.57 | 0.60 |

**Notes:** N=5,848,394 person-quarters. The denominator includes all eligible HIV Medicaid enrollees. The numerators for each condition include all patients who had evidence of the condition in the last 24 months, as documented in claims. OR is odds ratio, as derived from the logistic regression model, where an OR>1 implies that more enrollees had evidence of that condition in 2012 as compared to 2003. Coeff is coefficient, as derived from the linear regression model, where coeff>0 implies that enrollees had more comorbid conditions in 2012 as compared to 2003.

^a^ adjusted for age, sex, race, disability status, managed care status, state, and months of eligibility in the past 24 months. Unadjusted estimates are only adjusted for months of eligibility in the past 24 months.

**Figure A2a-k.** Unadjusted Trends in Proportion of Patients with Comorbid Condition with 95% Confidence Intervals, 2003-2012

A. B.

C. D.

E. F.

G. H.
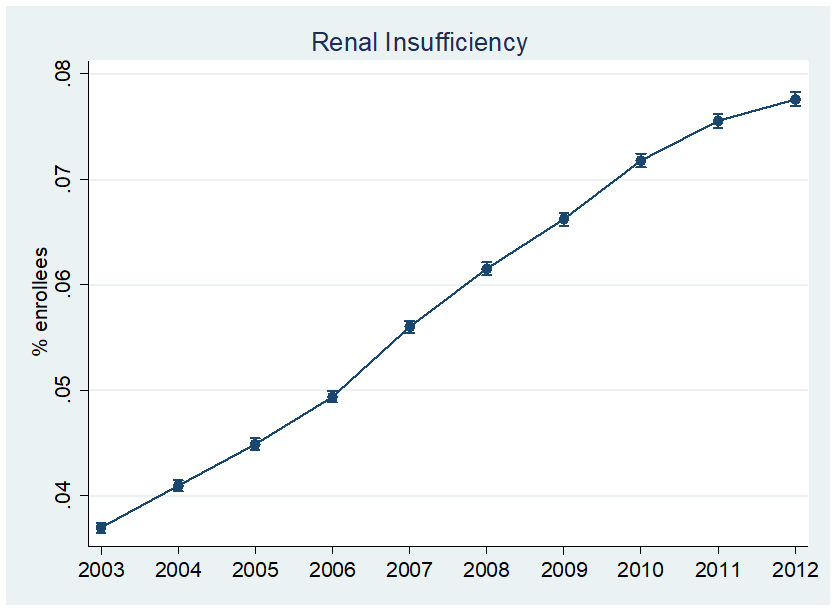


I. J.

K.

**Notes, A-K:** N=5,484,394 person-quarters. All yearly estimates have p<0.001. The denominator includes all eligible HIV Medicaid enrollees in the analytic cohort. The numerators for each condition include all patients who had evidence of the condition in the last 24 months, as documented in claims. Rates are adjusted for months of eligibility and not adjusted for patient characteristics.

**Table A5.** Unadjusted Percent of HIV Medicaid Enrollees with Comorbid Conditions, 2003-2012

| **Condition** | **2003** | **2004** | **2005** | **2006** | **2007** | **2008** | **2009** | **2010** | **2011** | **2012** | **All Years** |
| --- | --- | --- | --- | --- | --- | --- | --- | --- | --- | --- | --- |
| **Cardiovascular disease** | 16.9% | 17.8% | 18.5% | 19.1% | 19.5% | 19.4% | 19.4% | 19.5% | 19.4% | 19.3% | 18.9% |
| **Pulmonary disorder (any)** | 21.2% | 21.9% | 22.0% | 21.4% | 21.0% | 21.3% | 22.0% | 22.4% | 22.7% | 23.1% | 21.9% |
| **Hypertension** | 21.2% | 23.1% | 24.3% | 25.0% | 25.8% | 26.6% | 27.9% | 29.1% | 30.3% | 31.4% | 26.6% |
| **Diabetes** | 10.5% | 11.4% | 12.0% | 12.6% | 13.1% | 13.6% | 14.2% | 14.8% | 15.6% | 16.1% | 13.4% |
| **Liver disease (any)** | 19.0% | 20.9% | 21.9% | 22.2% | 22.3% | 22.5% | 23.2% | 23.8% | 24.5% | 25.4% | 22.6% |
| **Psychiatric disease** | 21.5% | 22.1% | 22.8% | 23.3% | 23.7% | 23.9% | 24.2% | 24.7% | 25.2% | 25.7% | 23.7% |
| **Hyperlipidemia** | 9.2% | 11.1% | 12.2% | 12.7% | 13.3% | 14.3% | 15.6% | 16.6% | 17.5% | 18.8% | 14.2% |
| **Anemia (most)** | 12.3% | 13.5% | 14.2% | 14.6% | 14.8% | 14.9% | 15.5% | 16.1% | 16.4% | 16.9% | 15.0% |
| **Renal insufficiency** | 3.7% | 4.1% | 4.5% | 4.9% | 5.6% | 6.2% | 6.6% | 7.2% | 7.6% | 7.8% | 5.8% |
| **Any HIV-related condition** | 15.8% | 15.8% | 15.4% | 14.8% | 14.6% | 14.5% | 14.2% | 13.6% | 13.1% | 12.9% | 14.4% |

**Notes:** N=5,848,394 person-quarters. All yearly estimates have p<0.001. The denominator includes all eligible HIV Medicaid enrollees in the analytic cohort. The numerators for each condition include all patients who had evidence of the condition in the last 24 months, as documented in claims. Rates are adjusted for months of eligibility and not adjusted for patient characteristics.

**Table A6.** Unadjusted percent of HIV Medicaid Enrollees with Evidence of 0-5+ Comorbid Conditions, 2003-2012

| **No. of Conditions** | **2003** | **2004** | **2005** | **2006** | **2007** | **2008** | **2009** | **2010** | **2011** | **2012** | **All Years** |
| --- | --- | --- | --- | --- | --- | --- | --- | --- | --- | --- | --- |
| 0 | 28.0% | 26.9% | 26.5% | 26.4% | 26.3% | 26.5% | 25.8% | 24.9% | 23.8% | 23.1% | 25.7% |
| 1 | 18.0% | 17.9% | 17.9% | 18.0% | 18.1% | 18.3% | 18.3% | 18.4% | 18.3% | 18.2% | 18.1% |
| 2 | 13.8% | 13.7% | 13.5% | 13.3% | 13.2% | 13.3% | 13.4% | 13.6% | 13.9% | 14.0% | 13.6% |
| 3 | 10.3% | 10.2% | 10.1% | 9.9% | 9.8% | 9.8% | 10.0% | 10.1% | 10.3% | 10.4% | 10.1% |
| 4 | 7.7% | 7.8% | 7.7% | 7.6% | 7.5% | 7.4% | 7.5% | 7.5% | 7.7% | 7.8% | 7.6% |
| 5+ | 22.3% | 23.6% | 24.4% | 24.9% | 25.0% | 24.8% | 25.0% | 25.7% | 26.1% | 26.5% | 24.9% |
| Mean no./enrollee | 2.74 | 2.87 | 2.94 | 2.99 | 3.01 | 3.00 | 3.04 | 3.12 | 3.18 | 3.22 | 3.02 |

**Notes:** N=5,848,394 person-quarters. All yearly estimates have p<0.001. The denominator includes all eligible HIV Medicaid enrollees in the analytic cohort. The numerators for each condition include all patients who had evidence of the number of conditions in the last 24 months, as documented in claims. Rates are adjusted for months of eligibility and not adjusted for patient characteristics.

# SUPPLEMENTAL CONTENT D: ADDITIONAL ANALYSES

**Table A7.** Changes in Comorbidities Amongst HIV Medicaid Enrollees with Clustering at State-level, 2012 vs 2003

|  | **Unadjusted** | | | | | | **Adjusted^a^** | | | | | |
| --- | --- | --- | --- | --- | --- | --- | --- | --- | --- | --- | --- | --- |
|  | **OR** | **Robust SE** | **z** | **p>\|z\|** | **[95% Conf.**  **Interval]** | | **OR** | **Robust SE** | **z** | **p>\|z\|** | **[95% Conf.**  **Interval]** | |
| **Cardiovascular disease** | 1.18 | 0.13 | 1.54 | 0.123 | 0.96 | 1.46 | 1.03 | 0.11 | 0.25 | 0.802 | 0.83 | 1.27 |
| **Pulmonary disorder (any)** | 1.12 | 0.05 | 2.52 | 0.012 | 1.03 | 1.22 | 1.05 | 0.05 | 1.06 | 0.290 | 0.96 | 1.16 |
| **Hypertension** | 1.72 | 0.08 | 11.58 | 0.000 | 1.57 | 1.88 | 1.31 | 0.11 | 3.07 | 0.002 | 1.10 | 1.55 |
| **Diabetes** | 1.65 | 0.13 | 6.29 | 0.000 | 1.41 | 1.93 | 1.26 | 0.08 | 3.61 | 0.000 | 1.11 | 1.44 |
| **Liver disease** | 1.45 | 0.12 | 4.65 | 0.000 | 1.24 | 1.70 | 1.31 | 0.12 | 2.99 | 0.003 | 1.10 | 1.57 |
| **Psychiatric disease** | 1.26 | 0.15 | 2.03 | 0.042 | 1.01 | 1.58 | 1.45 | 0.17 | 3.22 | 0.001 | 1.16 | 1.82 |
| **Hyperlipidemia** | 2.30 | 0.15 | 12.79 | 0.000 | 2.03 | 2.62 | 1.80 | 0.15 | 6.87 | 0.000 | 1.52 | 2.13 |
| **Anemia (most)** | 1.45 | 0.14 | 3.82 | 0.000 | 1.20 | 1.76 | 1.38 | 0.10 | 4.31 | 0.000 | 1.19 | 1.60 |
| **Renal insufficiency** | 2.20 | 0.11 | 15.11 | 0.000 | 1.99 | 2.44 | 2.20 | 0.10 | 17.32 | 0.000 | 2.01 | 2.40 |
| **Any HIV-related condition** | 0.78 | 0.04 | -5.10 | 0.000 | 0.71 | 0.86 | 0.82 | 0.04 | -4.24 | 0.000 | 0.75 | 0.90 |
|  | **Coef.** | **Robust SE** | **z** | **p>\|z\|** | **[95% Conf.**  **Interval]** | | **Coef.** | **Robust SE** | **z** | **p>\|z\|** | **[95% Conf.**  **Interval]** | |
| **No. of comorbid conditions** | 0.69 | 0.12 | 5.83 | 0.000 | 0.43 | 0.94 | 0.58 | 0.12 | 4.82 | 0.000 | 0.32 | 0.84 |

**Notes:** N=5,848,394 person-quarters. The denominator includes all eligible HIV Medicaid enrollees. The numerators for each condition include all patients who had evidence of the condition in the last 24 months, as documented in claims. OR is odds ratio, as derived from the logistic regression model, where an OR>1 implies that more enrollees had evidence of that condition in 2012 as compared to 2003. Coeff is coefficient, as derived from the linear regression model, where coeff>0 implies that enrollees had more comorbid conditions in 2012 as compared to 2003. All errors are clustered at the state-level. ^a^ adjusted for age, sex, race, disability status, managed care status, state, and months of eligibility in the past 24 months. Unadjusted estimates are only adjusted for months of eligibility in the past 24 months.

**Table A8.** Changes in Cancer Amongst HIV Medicaid Enrollees: HIV-related cancer vs. all other cancer, 2012 vs 2003

|  | **2003** | **2012** | **Unadjusted** | | | | | | **Adjusted** | | | | | |
| --- | --- | --- | --- | --- | --- | --- | --- | --- | --- | --- | --- | --- | --- | --- |
|  |  |  | **OR** | **Robust SE** | **z** | **p>\|z\|** | **[99% Conf.**  **Interval]** | | **OR** | **Robust SE** | **z** | **p>\|z\|** | **[99% Conf.**  **Interval]** | |
| **HIV-related cancer** | 3.2% | 2.4% | 0.74 | 0.05 | -4.49 | 0.000 | 0.65 | 0.84 | 0.85 | 0.05 | -2.61 | 0.000 | 0.76 | 0.96 |
| **Non-HIV-related cancer** | 3.2% | 4.8% | 1.54 | 0.20 | 3.32 | 0.001 | 1.19 | 1.9 | 1.35 | 0.22 | 1.85 | 0.064 | 0.98 | 1.85 |

**Table A9.** Changes in HIV-related Dementia Amongst HIV Medicaid Enrollees, 2012 vs 2003

|  | **2003** | **2012** | **Unadjusted** | | | | | | **Adjusted** | | | | | |
| --- | --- | --- | --- | --- | --- | --- | --- | --- | --- | --- | --- | --- | --- | --- |
|  |  |  | **OR** | **Robust SE** | **z** | **p>\|z\|** | **[99% Conf.**  **Interval]** | | **OR** | **Robust SE** | **z** | **p>\|z\|** | **[99% Conf.**  **Interval]** | |
| **HIV-related dementia** | 2.3% | 2.6% | 1.11 | 0.01 | 8.56 | 0.000 | 1.08 | 1.14 | 1.30 | 0.02 | 20.85 | 0.000 | 1.25 | 1.34 |

**Table A10.** Changes in Cancer Amongst HIV Medicaid Enrollees: Bone disorder, 2012 vs 2003

|  | **2003** | **2012** | **Unadjusted** | | | | | | **Adjusted** | | | | | |
| --- | --- | --- | --- | --- | --- | --- | --- | --- | --- | --- | --- | --- | --- | --- |
|  |  |  | **OR** | **Robust SE** | **z** | **p>\|z\|** | **[95% Conf.**  **Interval]** | | **OR** | **Robust SE** | **z** | **p>\|z\|** | **[95% Conf.**  **Interval]** | |
| **Bone disorder** | 2.4% | 4.0% | 1.71 | 0.17 | 5.28 | 0.000 | 1.40 | 2.08 | 1.38 | 0.10 | 4.42 | 0.000 | 1.20 | 1.59 |
